# Evaluating the effect of interventions for strengthening non-physician anesthetists’ education in Ethiopia: a pre- and post-evaluation study

**DOI:** 10.1186/s12909-021-02851-0

**Published:** 2021-08-08

**Authors:** Yohannes Molla Asemu, Tegbar Yigzaw, Firew Ayalew Desta, Fedde Scheele, Thomas van den Akker

**Affiliations:** 1 Jhpiego, An affiliate of Johns Hopkins University, Ethiopia country office, Addis Ababa, Ethiopia; 2 Athena Institute for Transdisciplinary Research, Amsterdam, The Netherlands; 3 OLVG Teaching Hospital, Amsterdam, The Netherlands; 4 Amsterdam UMC, Amsterdam, the Netherlands; 5 Dutch Royal Medical Council, Chair Legislative College for Accreditation of Residency Training 2016–2019, Utrecht, The Netherlands; 6 Athena Institute, Faculty of Science, Vrije Universiteit Amsterdam, Amsterdam, The Netherlands; 7 Department of Obstetrics and Gynaecology, Leiden University Medical Centre, Leiden, The Netherlands

**Keywords:** Anesthesia, Non-physician anesthetists, Ethiopia, Education quality, Objective structured clinical examination, Competence

## Abstract

**Background:**

Access to safe surgery has been recognized as an indispensable component of universal health coverage. A competent anesthesia workforce is a prerequisite for safe surgical care. In Ethiopia, non-physician anesthetists are the main anesthesia service providers. The Government of Ethiopia implemented a program intervention to improve the quality of non-physician anesthetists’ education, which included faculty development, curricula strengthening, student support, educational resources, improved infrastructure and upgraded regulations. This study aimed to assess changes following the implementation of this program.

**Methods:**

A pre-and post-evaluation design was employed to evaluate improvement in the quality of non-physician anesthetists’ education. A 10-station objective structured clinical examination (OSCE) was administered to graduating class anesthetists of 2016 (*n* = 104) to assess changes in competence from a baseline study performed in 2013 (*n* = 122). Moreover, a self-administered questionnaire was used to collect data on students’ perceptions of the learning environment.

**Results:**

The overall competence score of 2016 graduates was significantly higher than the 2013 class (65.7% vs. 61.5%, mean score difference = 4.2, 95% CI = 1.24–7.22, *p* < 0.05). Although we found increases in competence scores for 6 out of 10 stations, the improvement was statistically significant for three tasks only (pre-operative assessment, postoperative complication, and anesthesia machine check). Moreover, the competence score in neonatal resuscitation declined significantly from baseline (from 74.4 to 68.9%, mean score difference = − 5.5, 95% CI = -10.5 to − 0.5, *p* < 0.05). Initial gender-based performance differences disappeared (66.3% vs. 65.3%, mean score difference = − 1.0, 95% CI = − 6.11-3.9, *p* > 0.05 in favor of females), and female students scored better in some stations. Student perceptions of the learning environment improved significantly for almost all items, with the largest percentage point increase in the availability of instructors from 38.5 to 70.2% (OR = 3.76, 95% CI = 2.15–6.55, *p* < 0.05).

**Conclusion:**

The results suggest that the quality of non-physician anesthetists’ education has improved. Stagnation in competence scores of some stations and student perceptions of the simulated learning environment require specific attention.

## Background

Globally, health systems have undergone extensive changes over the past three decades. Promising opportunities are emerging while many barriers remain, particularly in low- and middle-income countries (LMIC). The number of health facilities with essential infrastructure has generally shown improvement along with the health workforce density. Key health outcome indicators including infant, under-five, and maternal mortality rates have all declined significantly worldwide. However, improvements are not uniform across nations, and LMICs continue to report higher mortality rates and a critical shortage of skilled health workforce [1]. In LMICs, morbidity, and mortality as a result of treatable surgical conditions have increased sharply while at the same time efforts made to improve access to safe and essential life-saving surgical and anesthetic care have stagnated. In LMICs an additional 143 million surgeries are required each year to save lives and prevent disability [2]. The Lancet Commission on Global Surgery estimates that, in Ethiopia alone, at least five million surgical procedures should be performed every year to meet population needs [3]. However, no more than 38,000 surgeries took place in 2012- less than 1% of the estimated need [4, 5].

While there is one physician anesthesia provider per 4000–5000 people in high-income countries, there is less than one provider per one million people in Ethiopia [6]. Such a critical shortage of the anesthesia workforce coupled with limited training capacity, varying training standards, and uneven distribution hinders efforts made by LMICs to ensure access to safe anesthetic care [6, 7]. The 2015–2020 health sector strategic plan made an unprecedented move by highlighting safe surgery and anesthesia as a priority area [8], responding to the World Health Assembly Resolution 68.15 on strengthening surgical and anesthetic care as part of ensuring Universal Health Coverage [9].

Given the critical shortage of physician anesthesia providers in the country, the Government of Ethiopia (GOE) scaled up anesthesia providers through a task-sharing that allowed non-physician clinicians to provide anesthetic services formerly reserved for medical specialists [8, 10, 11]. This workforce production was made possible through a rapid expansion of teaching institutions for non-physician anesthesia providers (also known as non-physician anesthetists) over seven years (2013–2020). As a result, non-physician anesthetists (NPAs) have become the predominant anesthesia service providers in Ethiopia and most east African countries [6, 8, 11, 12]. Despite reasonably similar clinical anesthesia roles, the training models for NPAs are heterogeneous in Ethiopia and across the LMICs in terms of their entry requirements, length of training, and qualifications awarded. In the African continent alone, there are about 22 different types of training models for NPAs across 51 countries [6, 13–15]. The entry requirements for these NPA training models were clinical nursing, other clinical experience, or high school completion in order of frequency. Diplomas and certificates were the widely awarded qualifications, followed by bachelor’s and master’s degrees with varying training durations from 9 months to 4 years depending on entry requirements and qualification awarded [13].

The Ethiopian training models for NPAs were originally designed to produce independent practitioners who can provide safe anesthetic care for essential and emergency surgical interventions. These models include a year of training for nurses (known as level-V anesthetic nursing), a 3-year baccalaureate training of nurses, a 4-year baccalaureate training program for high school graduates, and a 2-year post-baccalaureate master’s degree training [13, 16]. These models were carefully designed to allow career advancement of practitioners from the lowest level (Level-V anesthetic nursing), which is tasked to provide anesthetic care for the bellwether procedures to the baccalaureate and the master levels who relatively have broader practice scopes. The current Ethiopian strategic plan for surgery mainly focuses on the bellwether procedures (defined as cesarean section, laparotomy, and open fracture management) and few selected specialty services.

Improving anesthetic and surgical training and practice standards can contribute to at least nine of the 13 SDG3 targets directly or indirectly [17, 18]. Thus, it is imperative to periodically evaluate quality of anesthesia education by selecting suitable evaluation designs, like experimental and quasi-experimental studies [19]. By defining input, process and outcome parameters that quantify the main elements of an educational system, evaluation designs can be employed to assess changes in the quality of education [20]. In the medical education literature, input refers to the resources and infrastructure needed to run programs, while process refers to educational activities, and outcome embraces the measure of competence [21, 22]. However, evaluation data on the quality of training for the non-physician clinicians are scarce and marred with methodological limitations.

A 2013 baseline study conducted in Ethiopia showed significant gaps in the quality of NPA’s education [23]. The current study evaluated effects of program interventions by the GOE, the USAID Human Resources for Health (HRH) Project and other development partners. The primary study objective was to evaluate changes in the competence of graduating NPAs in 2016 students compared to the level in 2013. The secondary objective was to assess improvements in the learning environment over the three years.

## Methods

### Study design

Considering the complexity of health professionals’ education environment for tightly controlled designs with randomization, we opted for a quasi-experimental design [19]. To assess the impact of program interventions on quality of education we applied a pre-post evaluation design without control, implying data collection among different groups of learners who passed similar training programs [24]. Changes in competence of graduating NPAs and the learning environment from the baseline level were used as indicators to evaluate the quality of anesthesia education.

### Study population and setting

The study population was the graduating class NPAs in 4 public universities and 2 regional health science colleges (RHSCs). We decided to recruit all graduating class NPA students available at the time of the study. Participants from universities completed a baccalaureate degree program (clinical nurses and high school graduates) while those from RHSCs were clinical nurses with a year of additional training in anesthesia (level V anesthetic nurses). The two group study participants at the different institutions were following the same nationally harmonized curricula.

### Sample size and sample selection procedure

The evaluation employed a census at baseline and endline. All graduating students were invited to participate during the baseline (*n* = 200) and endline (*n* = 191) studies.

### Program interventions

The Lancet Commission on Global Surgery recommended investment in strengthening training and professional development to increase the availability and accessibility of anesthesia providers in LMICs [2]. The USAID HRH Project (2012–2019) supported efforts of the Government of Ethiopia to increase the availability and quality of NPAs and other health workers. Project interventions (Table 1) were designed based on the Preservice Education conceptual model developed by Johnson et al. [25]. The key intervention approaches were:

- Building the capacity of anesthesia faculty to enhance teaching and assessment skills;
- Strengthening curricula to ensure core competencies are clearly defined, measured, and attained;
- Strengthening clinical education to increase the development of core professional competencies;
- Improving student support system to improve retention and performance;
- Enhancing school infrastructure to create an enabling environment for learning;
- Strengthening quality improvement and regulatory systems; and
- Increasing engagement and capacity of local professional associations to maximize sustainability.

**Table 1:** Program interventions implemented between 2013 and 2016

Area	Elements of the program intervention	Timeline
Faculty Development	▪ Designed and delivered series of needs-based training for a total of 1434 anesthesia tutors & preceptors (double-counting might exist) ▪ Training areas included teaching methods (effective teaching skills, simulation-based training, problem-based learning, student assessment, instructional design skills) and technical updates on a variety of health topics	Since 2013
Curricula strengthening	▪ Developed four competency-based national curricula (Level V, post-basic BSc, generic BSc and MSc anesthesia programs) ▪ Objective Structured Clinical Examination (OSCE) and workplace-based assessments introduced	Since 2013
Clinical education	▪ Strengthened clinical practice sites with over 20 clinical management algorithms and 13 universal anesthesia machines integrated with patient monitors ▪ Donated 13 shuttle buses to facilitate clinical teaching	Since 2014
Student support system	▪ Gender awareness improved and gender-responsive learning strengthened ▪ Targeted and informed student selection and recruitment of female students strengthened ▪ Orientation and psychosocial counseling services provided ▪ Life skills training, tutorial classes and financial support provided to female students and other students with particular needs as identified by the respective teaching institution	Since 2013
Infrastructure	▪ Equipped skills development labs with 513 simulators ▪ Stocked medical libraries with 1174 anesthesia books and 8260 common books ▪ Supported the development and printing of 2405 Level V anesthesia training modules (13 types) ▪ Distributed electronic ICT resources, such as 12 laptops, 405 desktop computers and 24 LCD projectors.	Since 2014
Regulation	▪ Introduced national licensing examination ▪ Developed and supported the use of educational standards for self-review and accreditation purposes ▪ Developed code of ethics and conduct and scopes of practice for NPAs Developed nine continuing professional development (CPD) training packages	Since 2014
Sustainability	▪ Built leadership, strategic planning and management capabilities of the Ethiopian Association of Anesthetists (EAA) ▪ Opened and furnished an office with a CPD unit ▪ Purchased and donated a vehicle ▪ Increased membership from 230 to 2150	Since 2013

### Measures and instruments

Student competence was measured using a 10-station Objective Structured Clinical Examination (OSCE), a reliable and widely used method to assess clinical skills [26–29]. The stations were designed by trained subject matter experts to address core competencies required for the provision of safe anesthetic care for essential surgeries as identified by the World Bank [30]. To further ensure the content validity in the Ethiopian context, experts referred to local training curricula and crafted representative case scenarios and assessment rubrics encompassing the different competency domains. Eight of these stations were directly observed by a qualified assessor assigned to each station who exclusively used standardized objective checklists to rate performance. These stations covered [1] lumbar puncture [2]; neonatal resuscitation [3]; endotracheal intubation [4]; laryngeal mask airway insertion [5]; cardiopulmonary resuscitation [6]; chest examination [7]; preoperative screening assessment; and [8] routine anesthesia machine check. The remaining two stations required examinees to write down their answers and were later scored by assessors. These stations covered [1] interpreting postoperative complications; and [2] blood transfusion. Because of lack of mannequins and relevant educational resources, three stations (lumbar puncture, neonatal resuscitation, and anesthesia machine check) were unobservable in two of the study sites at baseline and improvised to an oral question. Scoring rubrics consisted of 5 to 16 items/steps that aimed to measure clinical decision making, communication, and psychomotor skills. Competence assessments were performed voluntarily for study purposes only.

To measure quality of the learning environment (inputs and processes) for the desired purpose, structured interviews were conducted with students. The interview consisted of 12 questions designed in line with the national educational program standard and the response options were ‘Yes’, ‘Partially’ or ‘No’ [31]. Furthermore, students were asked about the number of endotracheal intubations they performed during their study.

### Data collection

The assessment was administered by trained senior anesthesia instructors (12 at baseline and 16 at endline) who had experience with skills assessments. Assessors, both baseline and endline, received a 3-day training including role-plays and discussion of ethical issues a week immediately before the data collection schedule. Over half of the assessors recruited for the endline study had also participated in the baseline assessment. Those who participated in the baseline study assessed similar stations at the endline. Assessors were not assigned to institutions they served as teaching staff to reduce bias. Baseline data were collected in June and July 2013, while the endline assessments were done from June to September 2016.

Assessors first administered structured face-to-face interviews to collect data on sociodemographic characteristics and the number of endotracheal intubations performed during training, followed by posing 12 questions on their perceptions of the learning environment. Intubation is an index procedure monitored in the national NPAs’ curricula. Then the OSCE was administered. Students rotated through every OSCE station, read a case scenario and performed a task or responded to a question. Assessors observed the performance of each student to determine the successful conduct of each step listed in the observation checklist for that OSCE station. Ten minutes were allotted for a student to complete the task at each station. Overall data collection was overseen by trained supervisors.

### Data analysis

Data entry and cleaning were performed using CSPro 5.0 and analyzed in STATA 14. The normal distribution assumptions together with outlier, linearity, multicollinearity and homoscedasticity were checked before data analysis. Competence score was computed by calculating the percentage of steps that students performed satisfactorily at each OSCE station. Stations had equal weight and the mean of the 10 stations was calculated to determine overall competence score. An independent sample t-test was used to compare competence between baseline and endline. The percentage of students who replied “yes” to statements in the structured interview was calculated to indicate a positive perception of the learning environment. Those who replied “partly” were recoded as “no”, as the partial response is more likely to refer to a gap in the adequacy of the learning environment. The percentage of students who performed at least 200 endotracheal intubations was also calculated to check the attainment of the benchmark required in the Ethiopian curriculum. Also, the Odds ratios were computed using chi-square analysis to compare changes in perception of learning environment. A *p*-value of less than 0.05 was considered statistically significant for all tests.

### Ethical considerations

Ethical approval for the study was obtained from the Johns Hopkins Bloomberg School of Public Health Institutional Review Board (IRB #5051). Permission to conduct the study was also obtained from the Ministry of Health (MOH) and deans of training institutions. Informed oral consent was obtained from all study participants, and measures were taken to protect autonomy and data confidentiality.

## Results

### Characteristics of students

At baseline and endline, 122 and 104 graduating class students participated, respectively, which accounted for 61% (122/200) and 54.5% (104/191) of eligible students. University students were predominantly males and younger whereas college students were largely females and older. However, there were no statistically significant differences in sex, age, entry behavior or previous anesthesia-related job experience between baseline and endline study groups (Table 2).

**Table 2:** Comparison of non-physician anesthesia students’ sociodemographic characteristics, baseline (2013) and endline (2016)

Variable	University		***P***-value	RHSC		***P***-value
	Baseline (***n*** = 81)	Endline (=87)		Baseline (***n*** = 41)	Endline (***n*** = 17)	
**Gender**						
Male	69.1	57.47	0.117	21.95	23.53	0.574
Female	30.9	42.53		78.05	76.47	
**Age in years**						
20–24	88.9	96.55	0.051	17.07	29.41	0.510
25–30	11.1	3.45		70.73	58.82	
> = 30	0	0		12.2	11.76	
**Hometown**						
Urban	64.2	13.79	< 0.001*	58.54	35.29	0.107
Rural	35.8	86.21		41.46	64.71	
**Previous job experience related to anesthesia**						
Yes	4.9	4.7	0.606	4.88	17.65	0.144
No	95.1	95.4		95.12	82.35	
**Entry behavior**						
High school graduates	82.7	80.5	0.706	0	0	-^a^
Clinical nurses	17.3	19.5		100	100	

*Note: Abbreviation: RHSC, regional health science college*

P values were calculated using the Pearson chi-square test. * indicates P values considered statistically significant at < 0.05. ^*a*^ *indicates no staistics are computed because entry behavioue is constant*

### Changes in competence scores of students

**Fig. 1:**
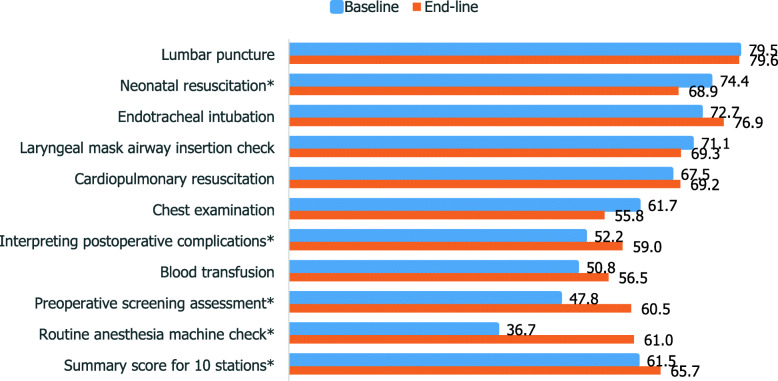
Non-physician anesthesia students’ mean competence scores, baseline (2013) and endline (2016). Abbreviation: OSCE, objective structured clinical examination. * indicates P values considered statistically significant at < 0.05

The mean score across all 10 OSCE stations revealed that the competence of students significantly increased by more than four percentage points from the baseline (65.7% vs. 61.5, 95% CI = 1.25–7.22, *p* < 0.05). Lumbar puncture for spinal anesthesia was the skill in which students scored the highest at endline (79.6%), followed by endotracheal intubation (76.9%). OSCE stations with the lowest competence scores at baseline showed a significant improvement at endline (Fig. 1).

As shown in Table 3, university students outperformed RHSC students at both baseline and endline, though the gap had widened from 9.0% at baseline to 18.6% at endline. University students scored significantly higher at endline than at baseline in three OSCE stations. On the contrary, students from universities scored significantly lower in laryngeal mask airway insertion at endline than at baseline (81.8% vs. 74.6%, mean score difference = − 7.2, 95%CI = − 12.7 to − 1.7, *p* < 0.05). Students from RHSCs had significantly lower scores in performing a lumbar puncture, neonatal resuscitation, and chest examination at endline compared to the baseline.

**Table 3:** Percentage change in the competence scores of non-physician anesthesia students from baseline (2013) to endline (2016), by institution type

OSCE station	University			RHSC		
	Baseline (*n* = 81)	Endline (*n* = 87)	Mean score difference [95% CI]	Baseline (*n* = 41)	Endline (*n* = 17)	Mean score difference [95% CI]
Lumbar puncture	78.9	82.8	+ 3.9 [−0.1, 8.7]	80.7	63.5	−17.2 [− 31.4, − 3.0]*
Neonatal resuscitation	75.3	72.2	−3.1 [−8.5, 2.3]	72.7	52.4	−20.3 [− 32.9, − 7.8]*
Endotracheal intubation	71.0	78.3	+ 7.3 [2.1, 12.6]*	76.2	69.5	−6.7 [− 18.0, 4.6]
Laryngeal mask airway insertion check	81.8	74.6	−7.2 [−12.7, − 1.7]*	50.1	42.5	− 7.6 [− 27.1, 11.8]
Cardiopulmonary resuscitation	66.3	70.2	+ 3.9 [−2.7, 10.5]	69.8	64.2	−5.6 [−18.5, 7.2]
Chest examination	65.2	59.2	−6.0 [−13.0, 0.09]	54.7	38.7	−16.0 [−27.9, − 4.0]*
Interpreting postoperative complications	55.6	60.9	+ 5.3 [−0.1, 10.9]	45.5	49.4	+ 3.9 [−8.3, 16.1]
Blood transfusion	58.8	59.1	+ 0.3 [−5.6, 6.1]	35.1	43.4	+ 8.3 [−4.1, 20.7]
Preoperative screening assessment	50.6	63.9	+ 13.3 [8.0, 18.5]*	42.3	43.1	+ 0.8 [−9.0, 10.7]
Routine anesthesia machine check	41.4	66.2	+ 24.8 [18.3, 31.4]*	27.4	34.3	+ 6.9 [−1.4, 15.2]
Summary score for 10 stations	64.5	68.7	+ 4.2 [1.49, 7.02]*	55.5	50.1	−5.4 [−12.7, 2.0]

*Abbreviations*: *OSCE* objective structured clinical examination, *RHSC* regional health science college

* *P* values considered statistically significant at < 0.05 as calculated by the independent sample t-test

Gender gaps in competence scores observed at baseline in favor of males (56.9% vs. 63.3%, mean score difference = 6.4, 95% CI = 2.6–10.0, *p* > 0.05) disappeared in the endline (66.3%vs. 65.3%, mean score difference = − 1.0, 95% CI = − 6.11-3.9, *p* > 0.05) (Data not shown).

### Students’ perceptions of the learning environment

Student perceptions of educational inputs and processes improved significantly from baseline for 11 of the 12 items studied, with the largest percentage point increase in the availability of instructors from 38.5 to 70.2% (OR = 3.76, 95% CI = 2.15–6.55, *p* < 0.05). Significant improvement was observed in almost all items at universities with the largest gain in the availability of instructors from 46.9 to 78.2% (OR = 4.05, 95% CI = 2.07–7.92, *p* < 0.05) (Table 4).

**Table 4:** Percent changes in non-physician anesthesia students’ perceptions of the learning environment from baseline (2013) to endline (2016), by training institution type

Conditions in facilities	Overall			University			RHSC		
	Baseline (*n* = 122)	Endline (*n* = 104)	OR [95% CI]	Baseline (*n* = 81)	Endline (*n* = 87)	OR [95% CI]	Baseline (41)	Endline (17)	OR [95% CI]
**Classroom**									
Number of instructors is adequate	38.5	70.2	3.76 [2.15, 6.55]*	46.9	78.2	4.05 [2.07, 7.92]*	21.9	29.4	1.48 [0.41, 5.32]
Instructors are effective in facilitating learning	50.0	67.3	2.06 [1.2, 3.54]*	41.9	70.1	3.24 [1.72, 6.13]*	65.9	52.9	0.58 [0.19, 1.84]
Instructors are fair and unbiased in assessing learning	48.4	71.0	2.3 [1.33, 3.96]*	34.6	64.4	3.42 [1.81, 6.45]*	75.6	88.2	2.42 [0.47, 12.45]
Classroom resources are available and helpful	26.2	47.1	2.51 [1.44, 4.38]*	27.2	47.1	2.39 [1.25, 4.56]*	24.4	47.1	2.76 [0.84, 9.05]*
**Skills lab**									
Number of skills lab assistants is adequate	4.9	21.4	5.25 [2.04, 13.53]*	2.5	25.6	13.58 [3.07, 59.9]*	9.8	0.0	0.02^a^*
Skills lab assistants effectively support students	9.0	33.7	5.12 [2.44, 10.74]*	6.2	35.6	8.4 [3.08, 23.0]*	14.6	23.5	1.8 [0.44, 7.4]
Skills lab resources are available and helpful	9.0	34.6	5.34 [2.55, 11.19]*	8.6	39.1	6.78 [2.79, 16.46]*	9.8	11.8	1.23 [0.2, 7.46]*
**Clinical practice sites**									
Clinical practice sites are conducive to learning	38.5	56.7	2.19 [1.28, 3.74]*	37.0	58.8	2.43 [1.3, 4.54]*	41.7	52.9	1.59 [0.51, 4.95]
Practical experience is sufficient	55.7	64.4	1.44 [0.84, 2.46]	58.0	69.0	1.61 [0.85, 3.03]	51.2	41.1	0.67 [0.21, 2.09]
Preceptors are present at practicum sites	72.1	91.3	4.08 [1.85, 8.99]*	62.9	90.8	5.81 [2.47, 13.67]*	90.2	94.1	1.73 [0.18, 16.72]
**Among students who said that preceptors were present at practicum sites:**									
Number of preceptors is adequate	25.4	45.2	1.73 [0.96, 3.14]*	31.4	55.0	2.67 [1.28, 5.59]*	41.7	18.8	0.32 [0.08, 1.33]
Preceptors are available at scheduled times and support students	33.6	55.8	1.68 [0.93, 3.02]*	38.0	58.8	2.25 [1.09, 4.65]*	59.5	68.8	1.5 [0.43, 5.21]

*Abbreviation*: *RHSC* regional health science college

* indicates *P* values considered statistically significant at < 0.05 by using the Pearson chi-square test

^a^ indicates a *P* value calculated using Fisher’s Exact Test as more than 25% of the cells have <5 observation

### Number of endotracheal intubations performed by students

The number of endotracheal intubations performed by the students during their training ranged widely both at baseline (20–601) and endline (28–350). While the overall median was 200 at both baseline and endline, university students performed more intubations than RHSC students at both baseline (220 vs. 95) and endline (200 vs. 65) (Data not shown).

## Discussion

Access to safe and affordable surgery depends partly on scaling up essential workforce density, addressing the critical shortage of human resources that has hampered service delivery to more than 5 billion people [2, 10, 32]. Task sharing is one of the most widely strategies used by LMICs to address the human resource gap [10, 32]. By expanding training of NPAs, the Ethiopian Government increased the anesthesia workforce density by more than fourfold - from 1 per 333,000 populations in 2012 [33] to 1 per 69,470 in 2019 [34]. However, resilient surgical and anesthetic care cannot be envisioned without addressing quality and safety concerns [18]. Particularly, when implementing task sharing, it is critical to maintain service quality and safety through human resource quality assurance mechanisms in areas of training and service provision [35–37].

A study we conducted in 2013 had documented large gaps in quality of non-physician anesthesia education [23]. The Government of Ethiopia implemented interventions to improve quality of education of health professionals. Thus, the objective of this study was to evaluate improvement in quality of anesthesia education. We found significant improvements not only in educational inputs and processes but also clinical competence of NPA students from the baseline. Considering the evaluation is carried out within a short period (1–3 years) after the interventions and during a time when the number and enrollment capacities of NPA teaching institutions are drastically increased, the observed improvement (especially in the university groups) is encouraging. However, the modest increment together with the stagnation or decline of some of the components assessed (e.g. neonatal resuscitation) raises concern about the preparation of these graduates to provide safe patient care. This could add fuel to the already increased anesthesia-related safety risk found in LMICs [18]. As data on the competence of NPAs is so scarce globally, comparison with similar studies was not possible. However, competence studies involving nurses [38, 39], midwives [40, 41], and other allied healthcare providers [42] across the LMICs have shown that acquiring sufficient competency levels is a global educational challenge.

With a positive shift in the university group, the overall improvement in the endotracheal intubation score in the OSCE (72.7% vs. 76.9%) is a positive achievement given that airway management is the number one safety issue in anesthesia practice [43] and the inability to perform it correctly contributes to about two-thirds of reported anesthesia-related maternal deaths [44]. In this study, the improvement in the intubation score despite a decline in the number of endotracheal intubations can be explained by the increased use of skill development labs (mannequins) to practice the skill during training [45, 46].

Findings from the endline assessment revealed that the pre-existing gender-based performance differences had disappeared, and even female students scored better in some stations. Targeted and informed student recruitment, motivation, and socioeconomic support of female students combined with institutional gender-responsive learning might have played a role in improving the performance of female students. Such transformative approaches are recommended by the WHO to improve gender equity in education [47]. Ensuring gender equality in health workforce training is important to achieve not only SDG 5 (gender equality) and SDG 3 (health and well-being), but also others including SDG 4 (quality education) and SDG 8 (decent work and inclusive economic growth) [48]. Collectively, this will bring significant health, social, and economic benefits to society [47, 49–51].

Of concern, students from RHSCs fall behind their university counterparts at both baseline and endline, gap widened at endline. Given that RHSC graduates are assigned after graduation to provide anesthesia service in primary hospitals where there is little to no opportunity to seek on-the-job assistance, efforts should be strengthened to improve the quality of this training. Critical faculty shortage, inadequate teaching facilities, and insufficient clinical practice opportunities to master core competencies may have contributed to the dismal finding. Increasing the use of simulation-based teaching, early clinical exposure, cognitive apprenticeship, and twining with nearby universities for shared resource utilization can be considered to address the competence gaps. Since this study did not explore other potential determinants of academic performance including high school grades and training duration, the recommendations have limitations.

The learning environment can have a lasting impact on the knowledge, critical reasoning, and motivation of the students. It serves as a key indicator for evaluating the curriculum and learning experiences of students where their satisfaction indicates the quality of an educational program [52–55]. This study revealed a significant improvement in the learning environment for almost all items studied, with the largest percentage point increases in the availability and effectiveness of instructors. This might be attributable to the government’s increased emphasis on availing a standard number of instructors along with the faculty development interventions implemented by the project. Besides, it is worth noting the efforts made by the project to strengthen the NPA MSc program may have improved availability of qualified instructors. However, the inadequate skill labs, limited classroom facilities, and poorly managed clinical practice were the persistent areas of concern. This finding calls for a wide-range of improvements including increasing the availability of teachers, skills lab assistants and clinical preceptors; increasing investment in learning environments, especially simulation-based teaching; implementing need-based faculty development programs; standardizing clinical learning; and enhancing public-private partnerships.

In summary, this study pointed out that task-sharing can be effectively employed to produce competent non-physicians who can provide clinical services originally designated for specialists. However, a well-delineated scope of practice that puts a delicate balance between access and quality of care is fundamental. Such a practice regulation is vital in preventing task-sharing that might result in uncontrolled and risky role modifications. The roles for different levels of practitioners should be set based on priority tasks, complexity and risk of procedures, and learning curve [10]. The learning curve can be enhanced by designing, implementing, and regulating need-based training. In this regard, education quality assurance, licensing, and accreditation systems are worth considering to standardize the quality of training nationally and regionally. Task-sharing shouldn’t be only seen as a short-term solution to fix specialist shortages, rather a sustainable and long-term approach that focuses on producing a workforce resilient to burnout and other professional challenges [36, 56–58]. Furthermore, it shouldn’t be late to pause and reflect on the different NPAs training models and establish an agreed-upon standardization framework.

### Strength and limitations

There are a range of strengths to this evaluation study. First, it covered all non-physician anesthesia training institutions to get nationally representative samples. Second, data were collected before and after an intervention, allowing evaluation of change in education quality between the two-time points. Third, multiple quality indicators were evaluated in the causal chain of educational inputs, processes, outputs, and outcome, providing a chance for triangulation. Finally, student performance was assessed using OSCE, which is the preferred method because of its high objectivity and reliability even in national licensing exams.

The most important limitations of this study are the lack of randomization and a control group. As a result, the evaluation design employed in this study might not distinguish the direct effects of the program interventions from any other effect due to external factors to the program [24, 59]. However, the authors have not identified and discovered any variations in program-related characteristics between the two groups. In this study, though assessors received extensive prior training to reduce variations in OSCE scoring, differences for inter-observer ratings cannot be avoided completely. This study didn’t compute inter-rater reliability to make comparisons between the baseline and endline groups. Two RHSCs assessed at the baseline were not part of the endline assessment since they did not have graduate class students during data collection. Thus, they were replaced by two others with similar characteristics. Finally, 78 students during baseline and 87 during endline did not participate in the study as they were not found on campus during data collection. Furthermore, variations in the performance scoring of three stations (lumbar puncture, neonatal resuscitation, and anesthesia machine check) at two sites (oral questioning during baseline vs. direct performance observation during the endline) might have contributed to the difference.

## Conclusion

The results of this study showed that the causal chain of educational inputs, processes, outputs, and outcomes have improved significantly since 2013, indicating better education quality of NPAs. Nonetheless, there were still considerable gaps suggesting the need to do more for greater and accelerated impact. Gaps include lack of progress in the competence of RHSC students, low absolute competence scores, and inadequate improvements in the learning environment in RHSCs.

Given that all students who participated in the study have joined the workforce, the study authors recommend that the Federal Ministry of Health and partners implement performance improvement measures such as on the job/ in-service training, mentoring, supportive supervision, audit, and feedback to enhance the skill of new graduates. Competencies with the lowest scores warrant examination of curricula and scopes of practice. Interventions at the pre-service education level shall focus on key challenges through innovative approaches, including the maximal use of educational technologies and digital learning.

While this study broke new ground in Ethiopia, it has important research implications as well. Future pre-post design evaluation studies should attempt to include a control group with an interrupted time-series design to better establish a relationship between cause and effect. The authors also recommend a mixed-methods study involving not only students but also teachers and academic leaders to support arguments that particular intervention can be related to the improvements in education quality.

## Data Availability

The dataset that supports the analysis and interpretation of findings from the current study are available from the corresponding author on reasonable request.

## Abbreviations

GOE: Government of Ethiopia
NPAP: Non-physician Anesthesia Providers
NPA: Non-physician Anesthetist
OSCE: Objective Structured Clinical Examination
RHSC: Regional Health Science College
